# Optimising carotegrast methyl use in ulcerative colitis: patient profiling, predictive biomarkers, and timing of efficacy evaluation (ASPECT study)

**DOI:** 10.1007/s00535-025-02299-9

**Published:** 2025-09-22

**Authors:** Katsuyoshi Matsuoka, Fumihito Hirai, Kenji Watanabe, Ryota Hokari, Taku Kobayashi, Masayuki Saruta, Hiroshi Nakase, Takahiro Suzuki, Gakuto Yamazaki, Toshifumi Hibi, Mamoru Watanabe, Tadakazu Hisamatsu

**Affiliations:** 1 Division of Gastroenterology and Hepatology, Department of Internal Medicine, Toho University Sakura Medical Center, 564-1, Shimoshizu, Sakura-Shi, Chiba 285-8741 Japan; 2 Department of Gastroenterology and Medicine, Fukuoka University Hospital, Fukuoka, Japan; 3 Department of Internal Medicine for Inflammatory Bowel Disease, University of Toyama, Toyama, Japan; 4 Department of Internal Medicine, National Defense Medical College, Tokorozawa, Japan; 5 Center for Advanced IBD Research and Treatment, Kitasato University Kitasato Institute Hospital, Tokyo, Japan; 6 Division of Gastroenterology and Hepatology, Department of Internal Medicine, The Jikei University School of Medicine, Tokyo, Japan; 7 Department of Gastroenterology and Hepatology, Sapporo Medical University School of Medicine, Sapporo, Japan; 8 EA Pharma Co., Ltd., Tokyo, Japan; 9 Kissei Pharmaceutical Co., Ltd., Tokyo, Japan; 10 Organoid Center, Graduate School of Medicine, Juntendo University, Tokyo, Japan; 11 Department of Gastroenterology and Hepatology, Kyorin University School of Medicine, Mitaka, Japan

**Keywords:** Carotegrast methyl, Ulcerative colitis, Calprotectin, Anti-integrin αvβ6, Leucine-rich α-2 glycoprotein

## Abstract

**Background:**

Carotegrast methyl is approved for treating patients with moderately active ulcerative colitis with inadequate response to 5-aminosalicylic acid agents. However, real-world evidence to guide optimal use is limited. This retrospective study aimed to characterise suitable patient profiles, identify biomarkers predictive of carotegrast methyl efficacy, and determine the appropriate timing for treatment evaluation.

**Methods:**

We analysed data from 186 patients enrolled in a phase 3 trial of carotegrast methyl (96 carotegrast methyl, 90 placebo). We assessed biomarkers (leucine-rich *α*-2 glycoprotein, C-reactive protein, faecal calprotectin, and anti-integrin αvβ6 antibody titres) alongside clinical outcomes and assessed symptom diaries and maintenance therapies.

**Results:**

Low disease activity at baseline (partial Mayo score ≤ 5) was associated with higher remission rates (56.1 vs. 33.3%). Anti-integrin αvβ6 antibody titres of < 44.6 U/mL at baseline predicted higher clinical remission rates (49.1 vs. 26.8%). Symptom improvement was detectable from week 2. Faecal calprotectin correlated with the endoscopic subscore at the end of treatment (*ρ* = 0.566); a faecal calprotectin level of < 387.5 µg/g predicted Mayo endoscopic subscore 0/1. Most patients maintained remission with oral 5-aminosalicylic acid alone; the 1-year maintenance rate was 56.5%.

**Conclusions:**

Carotegrast methyl appears most effective in patients with moderately active ulcerative colitis with low disease activity, with week 2 and beyond as an appropriate time point for assessing efficacy. Anti-integrin αvβ6 antibody titre shows promise in predicting response to carotegrast methyl. Faecal calprotectin may serve as a non-invasive surrogate for evaluating endoscopic healing.

**Supplementary Information:**

The online version contains supplementary material available at 10.1007/s00535-025-02299-9.

## Introduction

Ulcerative colitis (UC) is a chronic idiopathic disease characterised by recurrent episodes of remission and relapse, with symptoms, such as rectal bleeding, diarrhoea, and abdominal pain due to inflammation of the colorectal mucosa, from the rectum to the proximal colon [1]. In addition to causing abdominal symptoms, UC also burdens patients with other physical and mental challenges due to the anxiety, depression, reduced productivity, and decreased quality of life that are associated with this disease [2, 3].

Treatment for UC involves remission induction therapy during the active phase, followed by maintenance therapy to sustain remission. The typical treatment strategy uses both symptomatology and objective methods (such as endoscopic assessment, ultrasonography, and biomarkers) to establish treatment goals, evaluate treatment, and improve long-term outcomes [4]. For remission induction therapy, 5-aminosalicylic acid (5-ASA) agents are the first-line treatment, with corticosteroids being an option in severe patients. These 5-ASA agents remain the first-line treatment in remission maintenance therapy, with thiopurine agents considered in refractory patients [4].

Although the pathogenesis of UC is not fully understood [5], it is known that the inflamed areas of the gastrointestinal tract in affected patients are characterised by infiltration of inflammatory cells, including lymphocytes [5, 6]. Integrins—proteins that consist of an α chain and a *β* chain and are expressed on cell membranes, including in lymphocytes—are involved in binding to other cells and the extracellular matrix [5, 6]. In UC, there is overexpression of vascular cell adhesion molecule 1 (VCAM-1) and mucosal addressin cell adhesion molecule 1 (MAdCAM-1), which are adhesion molecules present on the surface of vascular endothelial cells in affected areas. This overexpression promotes the binding of lymphocytes to vascular endothelial cells via α4 integrins, thereby facilitating infiltration of inflammatory cells and exacerbating inflammation [6].

Carotegrast methyl (CGM), an α4 integrin inhibitor, is a therapeutic agent used to treat UC. The drug exerts anti-inflammatory effects by inhibiting both the binding of α4β1 integrins on inflammatory cells to VCAM-1 on vascular endothelial cells and the binding of α4β7 integrins to MAdCAM-1, thereby suppressing the extravasation of inflammatory cells and their accumulation in intestinal tissue [7]. In a phase 2 clinical trial [8] and phase 3 clinical trial (CT3 trial) [9] in Japanese patients with moderately active UC, treatment with CGM (CAROGRA^®^ Tablets; EA Pharma Co., Ltd., Tokyo, Japan) demonstrated superiority over placebo. In 2022, CGM was approved for clinical use in patients with moderately active UC who have had inadequate response to 5-ASA agents. It is not approved for use as remission maintenance therapy.

Ohmori reported that in a real-world study involving patients with UC, treatment with CGM resulted in significant reduction in the median Mayo endoscopic subscore (MES), from 3.0 to 0.0, and the median Mayo score, from 7.0 to 0.0 [10].

In recent years, various therapeutic agents with different pharmacological mechanisms have been approved for the treatment of UC, allowing for individualised treatment approaches. However, information remains limited regarding which types of patients are more likely to respond to each drug, including CGM. Data are also scarce on predictive factors of efficacy, timing of symptom improvement, biomarkers correlated with MES at the end of CGM treatment, and appropriate maintenance therapies for sustained remission.

With the aim of obtaining insights on how to optimise CGM treatment, this retrospective observational study investigated (i) characterisation of patients eligible for CGM treatment, (ii) baseline biomarkers to predict treatment efficacy, (iii) the temporal resolution of symptoms, (iv) biomarkers for predicting endoscopic severity at the end of CGM treatment for UC, and (iv) maintenance therapy after achievement of remission with CGM.

## Methods

### Study design

The CT3 trial was a multicentre, randomised, placebo-controlled, double-blind study of CGM in patients with insufficient response or intolerance to 5-ASA agents [9]. The present study, named the ASPECT study, was a retrospective observational study targeting patients enrolled in the CT3 trial, involving a total of 203 patients enrolled in the trial (102 patients from the CGM group and 101 patients from the placebo group). Biomarkers, including anti-integrin αvβ6 antibody, were measured from serum samples obtained in the CT3 trial. We reviewed daily symptom diaries from the CT3 trial and investigated medications used after CGM treatment.

### Assessments

Biomarkers were assessed using serum samples collected during the CT3 trial. Specifically, frozen serum samples were used to measure leucine-rich *α*-2 glycoprotein (LRG) and anti-integrin *α*v*β*6 antibody titres. LRG was measured for patients in the CGM and placebo groups. Anti-integrin αvβ6 antibody titres were measured for patients in the CGM group, using the Anti-Integrin αvβ6 ELISA Kit (Medical & Biological Laboratories Co., Ltd., Tokyo, Japan).

Stool frequency (Mayo stool frequency subscore) and the severity of rectal bleeding (Mayo rectal bleeding subscore) were assessed using data recorded daily by patients in symptom diaries. For patients who achieved clinical remission with CGM treatment, the names and doses of drugs used as remission maintenance therapy over the year following the end of CGM treatment were investigated. Clinical remission was defined as having a partial Mayo score of ≤ 2 and all subscores of ≤ 1. The data for LRG, anti-integrin αvβ6 antibody titres, daily symptom diaries, and drugs used as remission maintenance therapy were generated in this study; all other data utilised those from the CT3 trial.

### Endpoints

The endpoints of this study were (i) comparison of baseline patient characteristics between the groups with and without clinical remission at the end-of-treatment assessment; (ii) efficacy prediction based on biomarkers [LRG, C-reactive protein (CRP), faecal calprotectin (FCP), and anti-integrin αvβ6 antibody titres]; (iii) temporal comparisons of stool frequency (Mayo stool frequency subscore = 0/1) and resolution of rectal bleeding (Mayo rectal bleeding subscore = 0) between the CGM and placebo groups; (iv) cluster analysis of rectal bleeding resolution in the CGM group; (v) correlations between MES and levels of biomarkers at the end of treatment; additionally, for each biomarker, cut-off values for categorising MES = 0/1 and MES = 2/3, sensitivity, and specificity were calculated; (vi) clinical remission maintenance rates after achieving clinical remission with CGM treatment; and (vii) drugs used for maintenance therapy in patients who experienced clinical remission after treatment with CGM.

### Statistical analysis

Summary statistics were calculated for patient characteristics at baseline and indices. Comparisons of patient characteristics at baseline between those with clinical remission vs those without clinical remission were made using the Fisher’s exact test or Mann–Whitney U test. Using receiver-operating characteristic (ROC) curve analysis and the Youden index method, the predictive efficacy of biomarkers was evaluated by calculating cut-off values of the baseline biomarker levels to classify clinical remission and no clinical remission; sensitivity and specificity of the predictive biomarkers and clinical remission rates between patients with biomarker measurements below vs above the cut-off values were calculated. Stool frequency and resolution of rectal bleeding were assessed by day, and these endpoints were compared between the CGM and placebo groups using the Fisher’s exact test. Temporal changes in rectal bleeding resolution in the CGM group were analysed using a latent class mixed-effects model, and the optimal number of clusters was determined on the basis of the Bayesian information criterion. Correlations between biomarkers and MES at the end of treatment were evaluated using Spearman’s rank correlation coefficient (*ρ*). Comparisons between MES 0/1 and MES 2/3 groups were performed using the Wilcoxon’s rank sum test. ROC curve analysis was employed to calculate cut-off values for distinguishing between MES 0/1 and MES 2/3 using the Youden index method, and the sensitivity and specificity of these cut-off values were calculated. Clinical remission maintenance rates within 1 year after completing CGM treatment were estimated using the Kaplan–Meier method. For anti-integrin αvβ6 antibody titres exceeding the detection limit (200 U/mL), a value of 200.0 U/mL was imputed for the purpose of data analysis.

In all analyses, a two-sided *p* value of < 0.05 was considered statistically significant. Statistical analyses were performed using SAS Version 9.4 (SAS Institute Inc., Cary, NC, USA) and Microsoft Office 2010 (Microsoft Corporation, Redmond, WA, USA).

### Ethical considerations

Prior to their inclusion in the study, participants provided informed consent by written agreement or verbal agreement (with documentation) or were provided with the option to opt out. This study was approved by the Toho University Sakura Medical Center Ethics Committee (Approval No: S22048; approval date: 12 April 2023) and registered in the University Hospital Medical Information Network (UMIN) Clinical Trials Registry (UMIN000051508). The study has been conducted in accordance with the ethical standards laid down in the 1964 Declaration of Helsinki and its later amendments.

## Results

### Patient characteristics

Amongst the 203 patients enrolled in the CT3 trial, data were obtained from a total of 186 patients, including 96 patients in the CGM treatment group and 90 patients in the placebo group, which composed the population for data analysis in this study (Online Resource Figure S1). Data assessing rectal bleeding and stool frequency were available for 94 patients in the CGM group and 87 patients in the placebo group. Data on remission maintenance therapy were available for 48 patients, with a total of 59 data points, including patients who relapsed after the first course of CGM treatment and subsequently elected to enter into second and/or third CGM treatment courses. Table 1 summarises the patient characteristics of the CGM and placebo groups at baseline. The proportion of males was 63.5% in the CGM group and 61.1% in the placebo group. The median age was 43.0 years in the CGM group and 43.5 years in the placebo group. The median Mayo score was 8.0 in both groups. There were no significant differences in baseline characteristics between the two groups.

**Table 1:** Patient characteristics at baseline

	CGM group (*N* = 96)	Placebo group (*N* = 90)
Sex, *n* (%)		
Male	61 (63.5)	55 (61.1)
Female	35 (36.5)	35 (38.9)
Age, years, median [IQR]	43.0 [32.3–55.5]	43.5 [33.0–55.0]
Body weight, kg, median [IQR]	62.20 [54.53–71.30]	61.85 [54.20–68.08]
Disease duration, years, median [IQR]	5.0 [1.872–10.478]	3.4 [1.400–9.730]
Inadequate response/intolerance to 5-ASA agents^a^ or corticosteroids, *n* (%)		
Inadequate response to 5-ASA agents	89 (92.7)	82 (91.1)
Intolerance to 5-ASA agents	7 (7.3)	8 (8.9)
Inadequate response to corticosteroids	6 (6.3)	9 (10.0)
Intolerance to corticosteroids	1 (1.0)	3 (3.3)
Disease extent, *n* (%)		
Pancolitis	36 (37.5)	35 (38.9)
Left-sided colitis	45 (46.9)	43 (47.8)
Proctitis	15 (15.6)	12 (13.3)
Mayo score, median [IQR]	8.0 [6.0–9.0]	8.0 [7.0–9.0]
Partial Mayo score, median [IQR]	6.0 [4.0–7.0]	6.0 [5.0–7.0]
Stool frequency subscore, median [IQR]	2.0 [1.0–3.0]	2.0 [1.0–3.0]
Rectal bleeding subscore, median [IQR]	2.0 [1.0–2.0]	2.0 [1.0–2.0]
Endoscopic subscore, median [IQR]	2.0 [2.0–2.0]	2.0 [2.0–2.0]
Physician’s global assessment subscore, median [IQR]	2.0 [2.0–2.0]	2.0 [2.0–2.0]
LRG, µg/mL, median [IQR]	15.75 [13.50–19.38]	14.70 [11.50–19.00]
CRP, mg/dL, median [IQR]	0.165 [0.070–0.385]	0.150 [0.070–0.363]
FCP, µg/g, median [IQR]	965.50 [402.00–2857.50]	1180.00 [295.00–4390.00]
Anti-integrin αvβ6 antibody titre, U/mL, median [IQR]	33.30 [9.25–95.48]	–

*5-ASA* 5-aminosalicylic acid, *CGM* carotegrast methyl, *CRP* C-reactive protein, *FCP* faecal calprotectin, *IQR* interquartile range, *LRG* leucine-rich α-2 glycoprotein, *UC* ulcerative colitis

^a^Including sulfasalazine agents

### Comparison of patient characteristics at baseline between those with and without clinical remission in the CGM group

The baseline characteristics of patients with and without clinical remission in the CGM group (first treatment course) are presented in Table 2. Patients who experienced clinical remission had a significantly lower median Mayo score (*p* = 0.001), partial Mayo score (*p* < 0.001), stool frequency subscore (*p* = 0.002), and rectal bleeding subscore (*p* = 0.012) compared to those who did not. The clinical remission rate was 57.5% (23/40) amongst patients with a Mayo score of ≤ 7 and 26.8% (15/56) amongst those with a Mayo score of ≥ 8. Clinical remission rates based on the partial Mayo score were 56.1% (23/41) amongst patients with a score of ≤ 5 and 33.3% (15/45) amongst those with a score of ≥ 6. For all four biomarkers, the median values for patients with clinical remission were lower than those for patients without clinical remission; however, the differences were not statistically significant.

**Table 2:** Patient characteristics at baseline in the CGM treatment group (first treatment course), by those with vs without clinical remission at the end of treatment

	Clinical remission (*N* = 38)	No clinical remission (*N* = 58)	*p* value
Sex, *n* (%)			
Male	21 (55.3)	40 (69.0)	0.198^b^
Female	17 (44.7)	18 (31.0)	
Age, years, median [IQR]	43.5 [35.0–57.3]	43.0 [29.8–54.5]	0.235^c^
Body weight, kg, median [IQR]	42.6 [54.38–72.63]	62.1 [54.68–69.20]	0.770^c^
Disease duration, years, median [IQR]	6.1 [2.318–11.378]	4.5 [1.730–9.940]	0.321^c^
Disease extent, *n* (%)			
Pancolitis	12 (31.6)	24 (41.4)	0.636^b^
Left-sided colitis	20 (52.6)	25 (43.1)	
Proctitis	6 (15.8)	9 (15.5)	
Inadequate response/intolerance to 5-ASA agents^a^ or corticosteroids, *n* (%)			
Inadequate response to 5-ASA agents	37 (97.4)	52 (89.7)	0.238^b^
Intolerance to 5-ASA agents	1 (2.6)	6 (10.3)	0.238^b^
Inadequate response to corticosteroids	0 (0.0)	6 (10.3)	0.078^b^
Intolerance to corticosteroids	1 (2.6)	0 (0.0)	0.396^b^
Mayo score			
Median [IQR]	7.0 [6.0–8.0]	8.0 [7.0–9.0]	0.001^c^
≤ 7, *n* (%)	23 (60.5)	17 (29.3)	
≥ 8, *n* (%)	15 (39.5)	41 (70.7)	
Partial Mayo score			
Median [IQR]	5.0 [4.0–6.0]	6.0 [5.0–7.0]	< 0.001^c^
≤ 5, *n* (%)	23 (60.5)	18 (31.0)	
≥ 6, *n* (%)	15 (39.5)	40 (69.0)	
Stool frequency subscore			
Median [IQR]	2.0 [1.0–2.0]	3.0 [1.8–3.0]	0.002^c^
0, *n* (%)	3 (7.9)	4 (6.9)	
1, *n* (%)	14 (36.8)	10 (17.2)	
2, *n* (%)	13 (34.2)	11 (19.0)	
3, *n* (%)	8 (21.1)	33 (56.9)	
Rectal bleeding subscore			
Median [IQR]	1.0 [1.0–2.0]	2.0 [1.0–2.0]	0.012^c^
1, *n* (%)	22 (57.9)	19 (32.8)	
2, *n* (%)	14 (36.8)	31 (53.4)	
3, *n* (%)	2 (5.3)	8 (13.8)	
Endoscopic subscore, median [IQR]	2.0 [2.0–2.0]	2.0 [2.0–2.0]	0.855^c^
Physician’s global assessment subscore, median [IQR]	2.0 [2.0–2.0]	2.0 [2.0–2.0]	0.332^c^
LRG, µg/mL, median [IQR]	15.60 [13.18–18.65]	16.00 [13.50–21.55]	0.597^c^
CRP, mg/dL, median [IQR]	0.140 [0.068–0.345]	0.175 [0.078–0.420]	0.429^c^
FCP, µg/g, median [IQR]	776.00 [462.00–2700.00]	1020.00 [351.50–3785.00]	0.715^c^
Anti-integrin αvβ6 antibody titre, U/mL, median [IQR]	20.05 [6.25–84.38]	46.70 [15.58–100.30]	0.131^c^
Neutrophils,/µL, median [IQR]	3975.60 [2681.78–4737.08]	4009.60 [3002.90–5670.75]	0.231^c^
Eosinophils,/µL, median [IQR]	201.45 [143.85–353.65]	174.10 [79.80–382.05]	0.381^c^
Basophils,/µL, median [IQR]	30.60 [21.68–41.55]	32.25 [20.30–49.63]	0.779^c^
Lymphocytes,/µL, median [IQR]	1579.40 [1356.68–1797.45]	1556.30 [1272.00–1889.65]	0.653^c^
Monocytes,/µL, median [IQR]	363.85 [285.60–462.18]	424.70 [320.55–537.98]	0.085^c^

*5-ASA* 5-aminosalicylic acid, *CGM* carotegrast methyl, *CRP* C-reactive protein, *FCP* faecal calprotectin, *IQR* interquartile range, *LRG* leucine-rich α-2 glycoprotein, *UC* ulcerative colitis

^a^Including sulfasalazine agents

^b^Fisher’s exact test

^c^Mann–Whitney *U* test

No significant differences in other patient characteristics were observed between patients with and without clinical remission.

### Use of biomarkers to predict treatment efficacy

ROC analysis aimed at predicting clinical remission on the basis of biomarker levels at baseline yielded the following cut-off values (area under the curve [AUC] values): LRG = 18.6 µg/mL (0.532), CRP = 0.165 mg/dL (0.548), FCP = 692.5 µg/g (0.523), and anti-integrin αvβ6 antibody titre = 44.6 U/mL (0.591).

Clinical remission rates stratified by biomarker levels above and below the cut-off values are shown in Table 3. Rates of clinical remission were significantly higher amongst patients with a baseline anti-integrin αvβ6 antibody titre value of < 44.6 U/mL than amongst those with a baseline value of ≥ 44.6 U/mL (49.1% vs 26.8%; *p* = 0.035).

**Table 3:** Clinical remission rates by baseline biomarker levels (below vs above cut-off values) in patients receiving CGM treatment

	LRG, µg/mL				*p* value^a^
	< 18.6 (*N* = 65)		≥ 18.6 (*N* = 31)		
	*n*	%	*n*	%	
No clinical remission	36	55.4	22	71.0	0.183
Clinical remission	29	44.6	9	29.0	

	CRP, mg/dL				*p* value
	< 0.165 (*N* = 48)		≥ 0.165 (*N* = 48)		
	*n*	%	*n*	%	
No clinical remission	26	54.2	32	66.7	0.297
Clinical remission	22	45.8	16	33.3	

	FCP, µg/g				*p* value
	< 692.5 (*N* = 36)		≥ 692.5 (*N* = 54)		
	*n*	%	*n*	%	
No clinical remission	18	50.0	35	64.8	0.193
Clinical remission	18	50.0	19	35.2	

	Anti-integrin αvβ6 antibody titre, U/mL				*p* value
	< 44.6 (*N* = 55)		≥ 44.6 (*N* = 41)		
	*n*	%	*n*	%	
No clinical remission	28	50.9	30	73.2	0.035
Clinical remission	27	49.1	11	26.8	

Using ROC analysis, cut-off values were calculated for baseline biomarkers to classify clinical remission and nonclinical remission. Rates of clinical remission were then calculated for patients with biomarker values below and above these cut-off values

*CGM* carotegrast methyl, *CRP* C-reactive protein, *FCP* faecal calprotectin, *LRG* leucine-rich α-2 glycoprotein, *ROC* receiver-operating characteristic

^a^Fisher’s exact test

### Temporal changes in stool frequency and rectal bleeding resolution rates

**Fig. 1:**
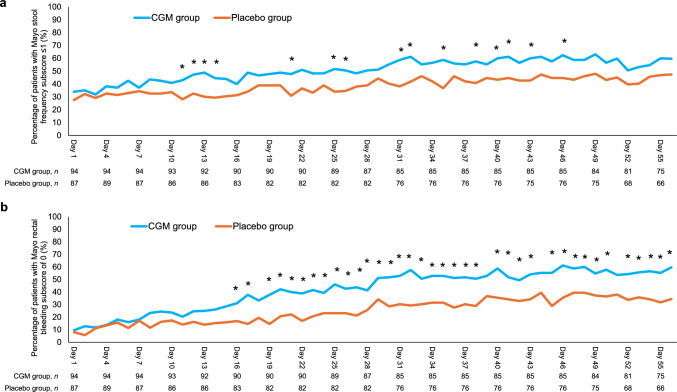
Temporal changes in stool frequency (**a**) and resolution of rectal bleeding (**b**). *CGM* carotegrast methyl. *p < 0.05; Fisher’s exact test

The percentage of patients with a Mayo stool frequency subscore of ≤ 1 differed significantly between the CGM and placebo groups beginning at day 11 after treatment initiation; at days 11–14, 21, 25–26, 31–32, 35, 38, 40–41, 43, and 46, this percentage in the CGM group was significantly higher than that in the placebo group (Fig. 1a). Similarly, the percentage of patients with a Mayo rectal bleeding subscore of 0 differed significantly between these groups beginning at day 16. Significant differences were observed at days 16–17, 19–38, 40–43, 45–50, and 52–56, with higher percentages of patients with a Mayo rectal bleeding subscore of 0 in the CGM group compared to the placebo group (Fig. 1b).

Regarding the Mayo rectal bleeding subscore in the CGM group, the distribution of scores after treatment is shown, stratified by baseline scores of 1 or ≥ 2 (Online Resource Figure S2). In patients with a baseline rectal bleeding subscore of 1, the proportion with a score of 0 was 50.0% at day 28, 55.6% at day 42, and 70.0% at day 56. In those with a baseline subscore of ≥ 2, the proportions with a score of 0 and 1, respectively, were 23.8% and 42.9% at day 28, 35.0% and 42.5% at day 42, and 42.9% and 31.4% at day 56.

### Cluster classification of temporal changes in rectal bleeding resolution

**Fig. 2:**
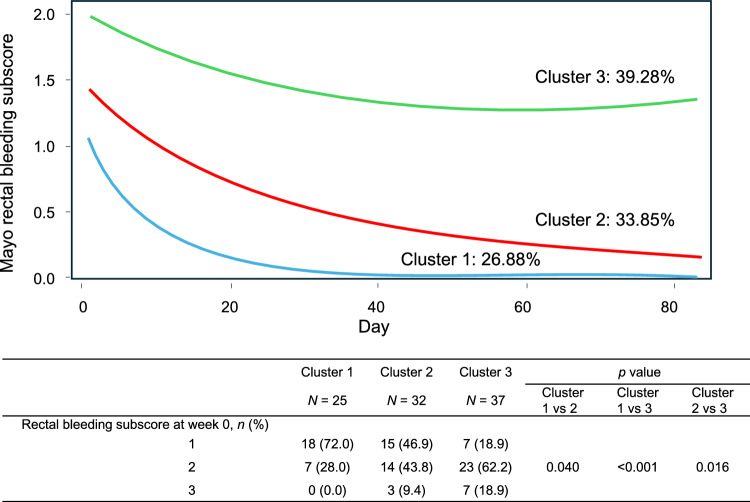
Temporal changes in resolution of rectal bleeding: cluster classifications. The percentages indicated above the curves show the predicted probabilities of classification for each cluster

Cluster analysis of rectal bleeding resolution in the CGM group identified three optimal clusters based on the Bayesian information criterion (Fig. 2). On the basis of their baseline rectal bleeding subscore, patients were classified into either cluster 1 (lowest rectal bleeding subscore), cluster 2 (intermediate rectal bleeding subscore), or cluster 3 (highest rectal bleeding subscore). Significant differences in baseline rectal bleeding subscore distributions were observed between clusters (cluster 1 vs cluster 2, *p* = 0.040; cluster 1 vs cluster 3, *p* < 0.001; cluster 2 vs cluster 3, *p* = 0.016). The predicted probabilities of classification were 26.88% for cluster 1, 33.85% for cluster 2, and 39.28% for cluster 3. Amongst patients with a Mayo rectal bleeding subscore of 1 at baseline, 82.5% (33/40) were classified into cluster 1 or cluster 2; amongst patients with a subscore of 2, 47.7% (21/44) were classified into cluster 1 or 2, and only 30% (3/10) of patients with a subscore of 3 were classified into these clusters.

### Correlation between MES and biomarkers at the end of treatment

In the CGM group, the correlation coefficients between biomarkers and MES at the end of treatment were as follows: LRG, *ρ* = 0.290 (*p* = 0.048); CRP, *ρ* = 0.269 (*p* = 0.071); FCP, *ρ* = 0.566 (*p* < 0.001); and anti-integrin αvβ6 antibody titre, *ρ* = 0.249 (*p* = 0.091) (Online Resource Table S1). Amongst CGM-treated patients, median FCP levels were significantly lower in the MES 0/1 subgroup compared to the MES 2/3 subgroup (109.5 µg/g vs 942.0 µg/g; *p* < 0.001) (Table 4). ROC analysis identified an FCP cut-off value of 387.5 µg/g for predicting MES 0/1, with a sensitivity of 92% and specificity of 63%. In the CGM group, no significant differences in the median levels of LRG, CRP, or anti-integrin αvβ6 antibody titres were observed between patients with MES 0/1 and those with MES 2/3.

**Table 4:** Biomarker values and MES classification at the end of CGM treatment: ROC analysis

	MES classification			ROC analysis			
	0/1	2/3	*p* value^a^	AUC	Cut-off value	Sensitivity	Specificity
CGM group							
LRG, µg/mL							
*n*	26	21					
Median [IQR]	13.75 [11.50–14.95]	17.60 [12.60–23.10]	0.053	0.666	17.0	85%	57%
CRP, mg/dL							
*n*	26	20					
Median [IQR]	0.085 [0.038–0.225]	0.160 [0.060–0.468]	0.258	0.598	0.155	69%	55%
FCP, µg/g							
*n*	26	19					
Median [IQR]	109.5 [31.2–239.0]	942.0 [214.0–3510.0]	< 0.001	0.853	387.5	92%	63%
Anti-integrin αvβ6 antibody titre, U/mL							
*n*	26	21					
Median [IQR]	22.00 [5.88–74.53]	62.50 [37.75–109.55]	0.066	0.658	40.6	65%	76%

*AUC* area under the curve, *CGM* carotegrast methyl, *CRP* C-reactive protein, *FCP* faecal calprotectin, *IQR* interquartile range, *LRG* leucine-rich α-2 glycoprotein, *MES* Mayo endoscopic subscore, *ROC* receiver-operating characteristic

^a^Wilcoxon’s rank sum test

The correlation coefficients between biomarkers and MES at the end of treatment in the overall analysis population and the placebo group are shown in Online Resource Table S1. The MES classification and ROC analysis for each biomarker in the overall analysis population and the placebo group are shown in Online Resource Table S2.

### Clinical remission maintenance rates up to 1 year after completing treatment

**Fig. 3:**
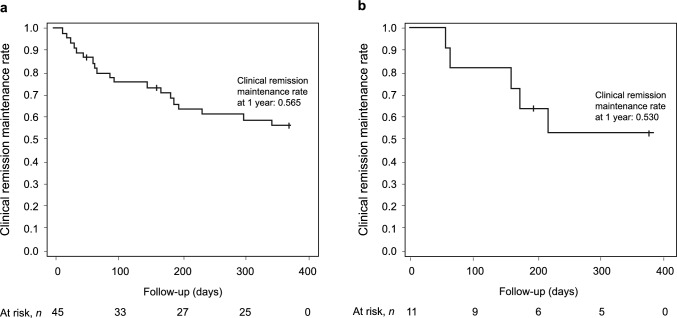
Clinical remission maintenance rates over the 1 year following the end of CGM treatment. Clinical remission maintenance rates amongst patients who achieved clinical remission after the first (**a**) and second (**b**) courses of CGM treatment. *CGM* carotegrast methyl

The clinical remission maintenance rates were 56.5% at 1 year (365 days) after completion of the first course of CGM treatment and 53.0% at 1 year after completion of the second course of CGM treatment (Fig. 3).

### Remission maintenance therapy after CGM treatment

For 48 patients who achieved clinical remission with CGM treatment, the drugs used in remission maintenance therapy were assessed at 59 points in time, including during the second and third treatment courses. The 59 recorded data points on maintenance therapy regimens included the following: use of monotherapy with an oral 5-ASA agent (*n* = 46), combination therapy with two oral 5-ASA agents (*n* = 3), a combination of oral 5-ASA agents and probiotics (*n* = 3), a combination of oral 5-ASA and enema 5-ASA (*n* = 1), and no maintenance therapy (*n* = 6). There was no use of immunomodulators, Janus kinase inhibitors, or biologics. The oral 5-ASA agents used in these maintenance therapy regimens included Multi-Matrix System formulations (*n* = 27), time-dependent formulations (*n* = 18), pH-dependent formulations (*n* = 6), and sulfasalazine (*n* = 5). At the start of remission maintenance therapy regimens, the mean ± standard deviation dose of oral 5-ASA agents was 4402 ± 636 mg. During the maintenance period, the dose of oral 5-ASA agents was reduced in two patients, and a short-term steroid enema foam was additionally prescribed for one patient.

## Discussion

In the CT3 trial, the improvement rate at 8 weeks after administration (the primary endpoint) was 45% in the CGM group and 21% in the placebo group, and the endoscopic healing rate (MES 0/1) was 55% in the CGM group and 27% in the placebo group, with a statistically significant difference observed between the two groups [9]. When seen from another perspective, these results also indicate that amongst patients in the CGM group, 55% did not achieve the primary endpoint and 45% did not achieve MES 0/1. Therefore, identifying patient populations with a high response rate to CGM is important for advancing personalised medicine.

In this study (ASPECT), with the aim of obtaining insights on how to optimise the use of CGM, we investigated not only suitable patient profiles and predictive factors of efficacy but also the timing of symptom improvement, biomarkers correlated with MES at the end of treatment, and appropriate maintenance therapies for sustained remission.

Amongst the analysed biomarkers, LRG, CRP, and FCP have been validated for assessing disease activity in UC and are widely used in clinical practice [11–15]. Additionally, anti-integrin αvβ6 antibody titre has been reported to have high sensitivity and specificity for diagnosing UC and is useful in evaluating disease activity [16, 17].

In the CGM group in this study, patients who achieved clinical remission in the first course of treatment had significantly lower median values for multiple endpoints than did patients with no clinical remission in this treatment course, as seen in the Mayo score (7.0 vs 8.0; *p* = 0.001), partial Mayo score (5.0 vs 6.0; *p* < 0.001), stool frequency subscore (2.0 vs 3.0; *p* = 0.002), and rectal bleeding subscore (1.0 vs 2.0; *p* = 0.012). Clinical remission rates were high in patients with a partial Mayo score of ≤ 5. Similarly, rates of clinical remission by rectal bleeding subscore were 20.0% (2/10) amongst patients with a score of 3, 31.1% (14/45) amongst those with a score of 2, and 53.7% (22/41) amongst those with a score of 1. Based on these results, CGM treatment appears to be most effective in patients who have moderate UC with relatively mild symptoms (partial Mayo score ≤ 5). The median values of all four biomarkers were lower in patients with clinical remission compared to those without clinical remission, although these differences were not statistically significant. The difference in median anti-integrin αvβ6 antibody titres between these patient groups, however, was relatively large, and the rates of clinical remission differed significantly between patients with values of this biomarker that were below the ROC analysis cutoff and those whose biomarker values were above the cutoff. Unlike the other three biomarkers, anti-integrin αvβ6 antibodies are not produced during inflammation but rather act as autoantibodies that inhibit the binding of proteins involved in colonic epithelial adhesion [16]. Higher levels of this autoantibody may be associated with responsiveness to CGM treatment, although the exact mechanism remains unclear. The relationship between anti-integrin αvβ6 antibody, the pathophysiology of UC, and drug treatment remains to be further elucidated. To our knowledge, no previous studies have investigated anti-integrin αvβ6 antibody titres as a predictive marker of therapeutic efficacy. Thus, a comparison of our results with existing studies and external validation of the cut-off value derived from the ROC analysis were not feasible.

Amongst patients in this study, stool frequency and the rate of rectal bleeding resolution differed significantly between the CGM and placebo groups from approximately the second week after treatment initiation. These findings suggest that it is appropriate to evaluate CGM treatment efficacy based on subjective symptoms from the second week of treatment onward.

Cluster analysis of the rectal bleeding score was used to classify patients into three clusters: cluster 1 (early improvement), cluster 2 (intermediate improvement), and cluster 3 (poor improvement). Most patients with a Mayo rectal bleeding score of 1 or 2 were classified into cluster 1 or 2. This finding, along with the reported clinical remission rates by rectal bleeding subscore before treatment initiation (Table 2), suggests that from the perspective of rectal bleeding improvement, patients with a Mayo rectal bleeding subscore of 1 or 2 may be suitable candidates for CGM treatment.

Colonoscopy is the current gold standard for monitoring endoscopic healing in patients with UC, but this procedure is invasive, time-consuming, and challenging to perform repeatedly.

In this study, we examined several biomarkers and their cut-off values for predicting MES at the end of CGM treatment. Yoshida et al. reported biomarker cut-off values for predicting MES 0/1, including an LRG value of 16.3 µg/mL and a CRP value of 0.18 mg/dL in patients with UC [13]. In the present study, biomarker cut-off values for MES 0/1 in the CGM group were LRG = 17.0 µg/mL (AUC, 0.666; sensitivity, 85%; specificity, 57%) and CRP = 0.155 mg/dL (AUC, 0.598; sensitivity, 69%; specificity, 55%). These cut-off values were comparable to those reported by Yoshida et al. [13], although the sensitivity and specificity values in our study were slightly lower than those in the comparable study.

Regarding the correlation coefficients between clinically used biomarkers and MES, Yoshida et al. [13] reported that the correlation coefficient for LRG was *ρ* = 0.424 (*p* < 0.001) and *ρ* = 0.459 (*p* < 0.001) for CRP; Lee et al. [18] reported a correlation coefficient of *ρ* = 0.304 (*p* < 0.0001) for FCP. In our study, the correlation coefficients were *ρ* = 0.290 (*p* = 0.048) for LRG, *ρ* = 0.269 (*p* = 0.071) for CRP, and *ρ* = 0.566 (*p* < 0.001) for FCP, with FCP showing the strongest correlation.

Anti-integrin αvβ6 antibody titres were not significantly correlated with the MES in the CGM group of the present study (*ρ* = 0.249; *p* = 0.091). However, we cannot comment on these data in relation to the existing literature, given that, to the best of our knowledge, no published studies have evaluated the correlation between anti-integrin αvβ6 antibody titres and MES. Collectively, our results suggest that FCP may be the most useful biomarker for predicting MES 0/1 at the end of CGM treatment. These findings warrant further investigation, since the use of biomarkers as a potential replacement for endoscopic evaluation in patients undergoing CGM treatment for UC could significantly reduce the burden on this patient population.

This study investigated remission maintenance therapy regimens at 59 data points in 48 patients who achieved clinical remission with CGM treatment; nearly 80% of these regimens involved monotherapy with oral 5-ASA agents. Although the combinations of oral 5-ASA agents with immunomodulators could also be considered as maintenance therapy after CGM, no such regimens were observed in this study.

Finally, a limitation of this study is that in the utilised data, some patients were missing data on MES at the end of treatment. Patients with such missing data were excluded from the data set used to evaluate relationships between biomarkers and MES, resulting in a limited sample size for these analyses.

## Conclusions

The findings of this study suggest that CGM is most suitable as treatment in patients who have moderate UC with low disease activity (partial Mayo score ≤ 5). Additionally, baseline anti-integrin αvβ6 antibody titre shows promise as a marker for predicting the efficacy of CGM treatment. Our results also indicate that evaluating the efficacy of CGM treatment based on improvement of subjective symptoms is appropriate from the second week of treatment and onward. Amongst the biomarkers evaluated in this study, FCP demonstrated the greatest potential as a non-invasive surrogate for evaluating endoscopic healing. To conclude, the findings of this study will help inform clinical decision-making when optimising CGM treatment for patients with UC.

## Supplementary Information

Below is the link to the electronic supplementary material.

Supplementary file1 (PDF 309 KB)
