# Correction: Optimising carotegrast methyl use in ulcerative colitis: patient profiling, predictive biomarkers, and timing of efficacy evaluation (ASPECT study)

**DOI:** 10.1007/s00535-026-02399-0

**Published:** 2026-04-10

**Authors:** Katsuyoshi Matsuoka, Fumihito Hirai, Kenji Watanabe, Ryota Hokari, Taku Kobayashi, Masayuki Saruta, Hiroshi Nakase, Takahiro Suzuki, Gakuto Yamazaki, Toshifumi Hibi, Mamoru Watanabe, Tadakazu Hisamatsu

**Affiliations:** 1 Division of Gastroenterology and Hepatology, Department of Internal Medicine, Toho University Sakura Medical Center, 564-1, Shimoshizu, Sakura-Shi, Chiba 285-8741 Japan; 2 Department of Gastroenterology and Medicine, Fukuoka University Hospital, Fukuoka, Japan; 3 Department of Internal Medicine for Inflammatory Bowel Disease, University of Toyama, Toyama, Japan; 4 Department of Internal Medicine, National Defense Medical College, Tokorozawa, Japan; 5 Center for Advanced IBD Research and Treatment, Kitasato University Kitasato Institute Hospital, Tokyo, Japan; 6 Division of Gastroenterology and Hepatology, Department of Internal Medicine, The Jikei University School of Medicine, Tokyo, Japan; 7 Department of Gastroenterology and Hepatology, Sapporo Medical University School of Medicine, Sapporo, Japan; 8 EA Pharma Co., Ltd., Tokyo, Japan; 9 Kissei Pharmaceutical Co., Ltd., Tokyo, Japan; 10 Organoid Center, Graduate School of Medicine, Juntendo University, Tokyo, Japan; 11 Department of Gastroenterology and Hepatology, Kyorin University School of Medicine, Mitaka, Japan

**Correction: Journal of Gastroenterology (2025) 60:1523–1534** 10.1007/s00535-025-02299-9

In the sentence beginning “Low disease activity at baseline…” in the Abstract under the heading Results, the value “33.3%” should have read “27.3%”. Furthermore, in the ‘Results’ section, in the sentence beginning “Clinical remission rates based on the…”, the value “33.3% (15/45)” should have read “27.3% (15/55)”.

