# Supplementary material for: Optimising carotegrast methyl use in ulcerative colitis: patient profiling, predictive biomarkers, and timing of efficacy evaluation (ASPECT study)
Source: J Gastroenterol. 2025 Sep 22;60(12):1523–34. doi: 10.1007/s00535-025-02299-9 (PMC12630152; doi:10.1007/s00535-025-02299-9)

Journal of Gastroenterology

Optimising Carotegrast Methyl Use in Ulcerative Colitis: Patient Profiling, Predictive Biomarkers, and Timing of Efficacy Evaluation (ASPECT Study)

Authors:
Katsuyoshi Matsuoka[1], Fumihito Hirai[2], Kenji Watanabe[3], Ryota Hokari[4], Taku Kobayashi[5], Masayuki Saruta[6], Hiroshi Nakase[7], Takahiro Suzuki[8], Gakuto Yamazaki[9], Toshifumi Hibi[5], Mamoru Watanabe[10], Tadakazu Hisamatsu[11]

Affiliations:
[1]Division of Gastroenterology and Hepatology, Department of Internal Medicine, Toho University Sakura Medical Center, Sakura, Japan
[2]Department of Gastroenterology and Medicine, Fukuoka University Hospital, Fukuoka, Japan
[3]Department of Internal Medicine for Inflammatory Bowel Disease, University of Toyama, Toyama, Japan
[4]Department of Internal Medicine, National Defense Medical College, Tokorozawa, Japan
[5]Center for Advanced IBD Research and Treatment, Kitasato University Kitasato Institute Hospital, Tokyo, Japan
[6]Division of Gastroenterology and Hepatology, Department of Internal Medicine, The Jikei University School of Medicine, Tokyo, Japan
[7]Department of Gastroenterology and Hepatology, Sapporo Medical University School of Medicine, Sapporo, Japan
[8]EA Pharma Co., Ltd., Tokyo, Japan
[9]Kissei Pharmaceutical Co., Ltd., Tokyo, Japan
[10]Organoid Center, Graduate School of Medicine, Juntendo University, Tokyo, Japan
[11]Department of Gastroenterology and Hepatology, Kyorin University School of Medicine, Mitaka, Japan

Corresponding author:
Katsuyoshi Matsuoka
Division of Gastroenterology and Hepatology, Department of Internal Medicine, Toho University Sakura Medical Center
Email: matsuoka@fk2.so-net.ne.jp

Table S1 Correlations between biomarkers and MES at the end of treatment

	Correlation between biomarker and MES	
**Treatment group**	***ρ***[a]	***p* value**
Overall (CGM and placebo groups)		
LRG ($n = 86$)	0.435	< 0.001
CRP ($n = 85$)	0.454	< 0.001
FCP ($n = 82$)	0.582	< 0.001
CGM group		
LRG ($n = 47$)	0.290	0.048
CRP ($n = 46$)	0.269	0.071
FCP ($n = 45$)	0.566	< 0.001
Anti-integrin αvβ6 antibody titre ($n = 47$)	0.249	0.091
Placebo group		
LRG ($n = 39$)	0.575	< 0.001
CRP ($n = 39$)	0.565	< 0.001
FCP ($n = 37$)	0.481	0.003

Abbreviations: CGM, carotegrast methyl; CRP, C-reactive protein; FCP, faecal calprotectin; LRG, leucine-rich α-2 glycoprotein; MES, Mayo endoscopic subscore.

[a] Spearman's rank correlation coefficient

Table S2 Biomarker values and MES classification at the end of CGM treatment (overall and placebo group): ROC analysis

	MES classification			ROC analysis			
	**0/1**	**2/3**	***p* value**[a]	**AUC**	**Cutoff value**	**Sensitivity**	**Specificity**
Overall (CGM and placebo groups)							
LRG, µg/mL							
n	38	48					
Median [IQR]	12.95 [10.55–14.65]	18.45 [12.38–24.53]	< 0.001	0.753	17.0	87%	63%
CRP, mg/dL							
n	38	47					
Median [IQR]	0.070 [0.030–0.190]	0.240 [0.100–0.720]	< 0.001	0.755	0.095	66%	81%
FCP, µg/g							
n	36	46					
Median [IQR]	109.5 [26.9–292.3]	937.5 [324.0–2982.5]	< 0.001	0.823	387.5	89%	72%
Placebo group							
LRG, µg/mL							
n	12	27					
Median [IQR]	10.65 [9.63–14.20]	18.80 [14.50–25.40]	< 0.001	0.866	15.5	92%	70%
CRP, mg/dL							
n	12	27					
Median [IQR]	0.045 [0.030–0.088]	0.290 [0.180–0.840]	< 0.001	0.906	0.095	83%	93%
FCP, µg/g							
n	10	27					
Median [IQR]	133.4 [12.2–926.0]	933.0 [413.0–2390.0]	0.015	0.763	339.5	80%	81%

Abbreviations: AUC, area under the curve; CGM, carotegrast methyl; CRP, C-reactive protein; FCP, faecal calprotectin; IQR, interquartile range; LRG, leucine-rich α-2 glycoprotein; MES, Mayo endoscopic subscore; ROC, receiver operating characteristic curve.

[a]Wilcoxon's rank sum test

Fig. S1 Flow chart of the study population

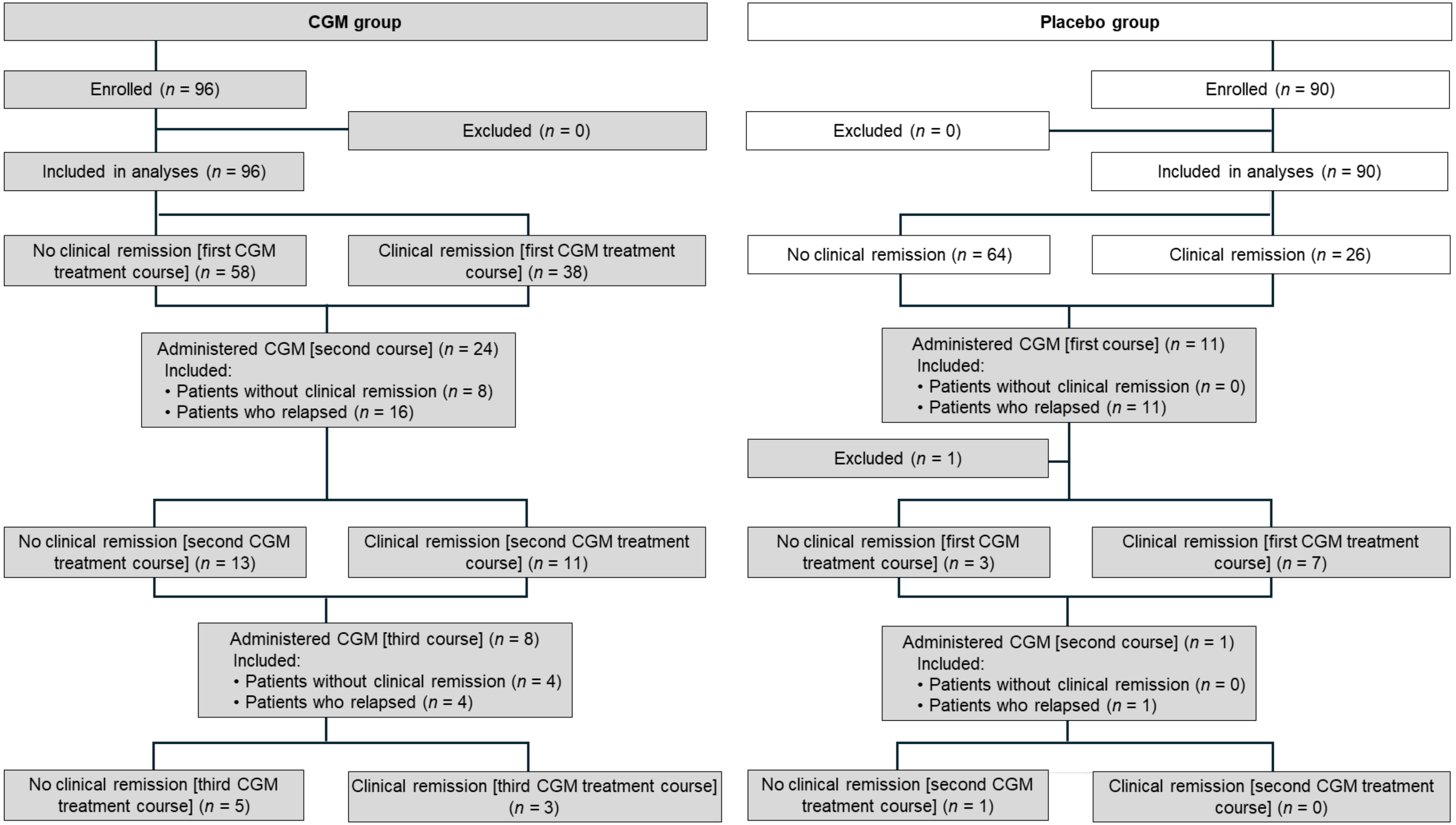

Abbreviation: CGM, carotegrast methyl.

Fig. S2 Distribution of Mayo rectal bleeding subscores after treatment in the CGM group, stratified by baseline scores of 1 or ≥ 2

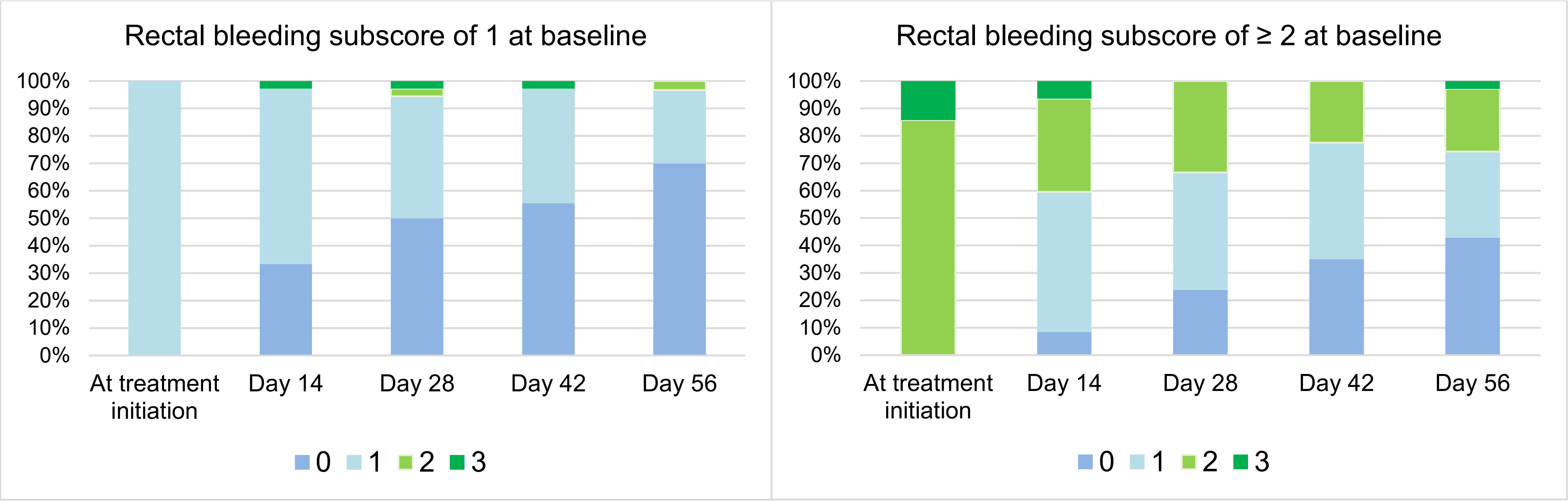

Supplement: Supplementary file 1 — Supplementary file1 (PDF 309 KB) [file 535_2025_2299_MOESM1_ESM.pdf]
